# Hyperpolarized ^129^Xe Time-of-Flight MR Imaging of Perfusion and Brain Function

**DOI:** 10.3390/diagnostics10090630

**Published:** 2020-08-25

**Authors:** Yurii Shepelytskyi, Francis T. Hane, Vira Grynko, Tao Li, Ayman Hassan, Mitchell S. Albert

**Affiliations:** 1 Chemistry and Materials Science Program, Lakehead University, 955 Oliver Rd., Thunder Bay, ON P7B 5E1, Canada; yshepely@lakeheadu.ca (Y.S.); vgrynko@lakeheadu.ca (V.G.); 2 Thunder Bay Regional Health Research Institute, 980 Oliver Rd., Thunder Bay, ON P7B 5E1, Canada; francishane@gmail.com; 3 Chemistry Department, Lakehead University, 955 Oliver Rd., Thunder Bay, ON P7B 5E1, Canada; tli@lakeheadu.ca; 4 Thunder Bay Regional Health Sciences Centre, 980 Oliver Rd., Thunder Bay, ON P7B 6V4, Canada; hassana@tbh.net; 5 Northern Ontario School of Medicine, 955 Oliver Rd., Thunder Bay, ON P7B 5E1, Canada

**Keywords:** hyperpolarized ^129^Xe, hyperpolarized time-of-flight MRI, perfusion imaging, hemodynamic response, fMRI

## Abstract

Perfusion measurements can provide vital information about the homeostasis of an organ and can therefore be used as biomarkers to diagnose a variety of cardiovascular, renal, and neurological diseases. Currently, the most common techniques to measure perfusion are ^15^O positron emission tomography (PET), xenon-enhanced computed tomography (CT), single photon emission computed tomography (SPECT), dynamic contrast enhanced (DCE) MRI, and arterial spin labeling (ASL) MRI. Here, we show how regional perfusion can be quantitively measured with magnetic resonance imaging (MRI) using time-resolved depolarization of hyperpolarized (HP) xenon-129 (^129^Xe), and the application of this approach to detect changes in cerebral blood flow (CBF) due to a hemodynamic response in response to brain stimuli. The investigated HP ^129^Xe Time-of-Flight (TOF) technique produced perfusion images with an average signal-to-noise ratio (SNR) of 10.35. Furthermore, to our knowledge, the first hemodynamic response (HDR) map was acquired in healthy volunteers using the HP ^129^Xe TOF imaging. Responses to visual and motor stimuli were observed. The acquired HP TOF HDR maps correlated well with traditional proton blood oxygenation level-dependent functional MRI. Overall, this study expands the field of HP MRI with a novel dynamic imaging technique suitable for rapid and quantitative perfusion imaging.

## 1. Introduction

Perfusion measurements can provide vital information about the homeostasis of an organ [1] and can therefore be used as biomarkers to diagnose cardiovascular [2,3], renal [4], and neurological [5,6,7] diseases. Currently, the most commonly used techniques to measure perfusion are ^15^O positron emission tomography (PET) [8,9,10], xenon-enhanced computed tomography (CT) [11,12], single photon emission computed tomography (SPECT) [3,8,13], and arterial spin labeling (ASL) magnetic resonance imaging (MRI) [1,14,15,16,17,18]. In addition, dynamic susceptibility contrast (DSC) and dynamic contrast enhanced (DCE) MRI are frequently used for perfusion imaging [19,20,21]. Although these techniques are well-established, each has some serious drawbacks. CT requires high-dose ionizing radiation, PET and SPECT rely on injection of radioactive contrast agents and the acquired PET images are of low resolution, and the signal-to-noise ratio (SNR) and contrast of ASL images is low. DSC and DCE MRI require injection of contrast agents, most of which contain gadolinium, which was recently associated with a certain amount of toxicity [22,23]. In addition, the most commonly used gadolinium-based contrast agents are uncapable of crossing the blood–brain barrier [24,25,26], which makes cerebral perfusion imaging with these agents more challenging.

Hyperpolarized (HP) xenon-129 (^129^Xe) MRI is a powerful MRI approach used mainly for lung imaging and for the study of lung disorders [27,28]. A hyperpolarized (HP) metastable state is produced by spin exchange optical pumping [29,30,31], and is characterized by up to 10^5^ larger longitudinal magnetization compared to thermal polarization [27]. Therefore, the magnetic resonance signal of HP nuclei can be up to 10^5^ times stronger than at thermal equilibrium. Due to the ability of HP ^129^Xe to dissolve in blood and travel to highly perfused organs, HP ^129^Xe MRI was recently used to study the brain [6,32,33,34] and kidneys [35]. The last achievements in the field of HP ^129^Xe MRI allowed the investigation of cerebral perfusion changes associated with Alzheimer’s disease [34] and stroke [6].

Since HP ^129^Xe dissolves in the blood [36] and has extreme sensitivity to chemical environments [27], it can be used as a contrast agent to study blood flow and conduct perfusion measurements in tissues. Since the HP state is a nonequilibrium, metastable state, the longitudinal magnetization is not restored by spin-lattice relaxation once a radiofrequency (RF) pulse irradiates the nuclei. After irradiation of a volume element containing HP ^129^Xe dissolved in tissue or blood with a 90° RF pulse, the HP state is completely destroyed and the dissolved HP ^129^Xe does not produce any significant amount of signal. If there is continuous flow into the volume of dissolved ^129^Xe, and if the MR measurement is conducted following a prescribed time delay (time-of-flight (TOF) time), the MR signal is determined mainly by the amount of ^129^Xe washed into the selected volume. We hypothesize that it is possible to measure the blood flow and tissue perfusion quantitatively by creating a dynamic imaging technique which measures the ^129^Xe signal evolution with change in TOF. The proposed perfusion imaging technique has the potential to open a new pathway for imaging and diagnostics of perfusion-related diseases.

In this proof-of-concept study, we develop a novel HP ^129^Xe time-of-flight perfusion imaging pulse sequence and evaluate the performance of this technique in vitro and in vivo. The first quantitative HP cerebral perfusion images were acquired using the proposed technique in healthy volunteers. Furthermore, the HP ^129^Xe TOF technique was used for hemodynamic response detection and was corroborated by conventional proton (^1^H) blood oxygenation level-dependent (BOLD) fMRI.

## 2. Materials and Methods 

### 2.1. Pulse Sequence Design

**Figure 1:**
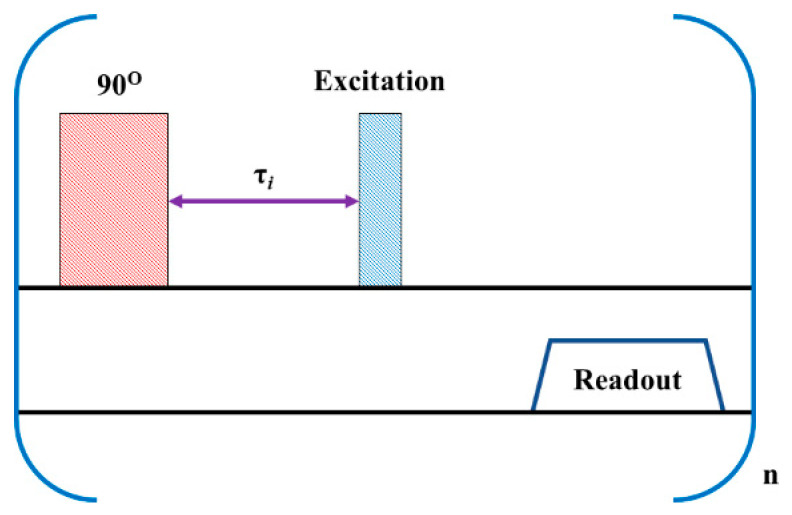
^129^Xe time-of-flight (TOF) perfusion imaging pulse sequence diagram. The 90° pulse with narrow bandwidth (in pink) is applied first. After the recovery time delay, τ (the purple arrow), the imaging pulse sequence is initiated. The excitation pulse is represented by the blue rectangle. This pulse sequence is repeated *n* times. Index i corresponds to the delay time with an index number i.

The Chemical Shift Saturation Recovery (CSSR) pulse sequence is typically used to study the time course of hyperpolarized (HP) ^129^Xe gas exchange in the lungs [37,38]. We modified the CSSR sequence for cerebral perfusion imaging using HP ^129^Xe to create the ^129^Xe TOF used in this study (Figure 1). The first 90° block pulse has a time duration of 0.5 ms, which yields a bandwidth (BW) of 2 kHz (56.5 ppm). This pulse is broad enough to saturate the HP ^129^Xe in all brain tissues and the Xe dissolved in blood. After termination of the saturation pulse, the unsaturated ^129^Xe from the lungs flows into the brain during time τ. The image pulse sequence is then initiated. Here, the broad-band excitation pulse of spredrex shape was used for imaging. The image was acquired using a Gradient Echo (GRE) pulse sequence with a Cartesian read-out. The TOF pulse sequence was repeated three times with different recovery times τ (Figure 1). To detect hemodynamic response to a visual stimulus, we used the following delay times: τ_0_ = 1 s, τ_1_ = 6.5 s, τ_2_ = 8 s. To detect hemodynamic response to a motor task, the TOF delays were equal to 2.5 s, 6.8 s, and 7.1 s. 

### 2.2. In Vitro Flow Measurements 

**Figure 2:**
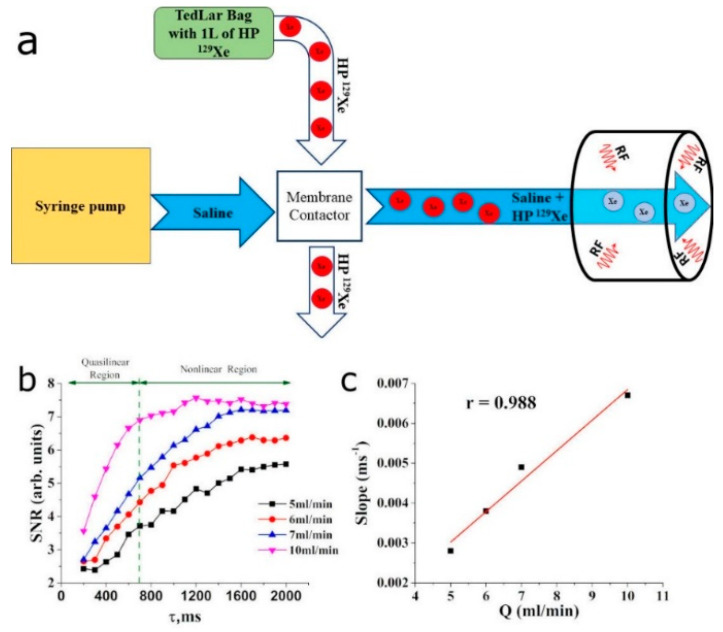
**In vitro phantom design and obtained time-of-flight (TOF) ^129^Xe curves with Pearson’s correlation coefficient.** (**a**) Schematic diagram of the flow phantom used in this study. A syringe pump provided four different flow rates, i.e., 5 mL/min, 6 mL/min, 7 mL/min, and 10 mL/ min. (**b**) ^129^Xe TOF recovery curves were measured using the TOF imaging approach. A difference in flow rate of 1 mL/min created well-detected differences in the TOF curves. A quasilinear region was observed between 200 ms and 700 ms. A nonlinear recovery appeared for longer recovery times, followed by a steady state. (**c**) Pearson’s linear correlation between the flow rate and the slopes of the TOF curves was calculated at the quasilinear region. A strong positive correlation (r ≈ 0.988) was observed, indicating the potential to use this slope for flow mapping.

The flow phantom used in this study is shown in Figure 2a. The 3.175 mm tube was connected to the syringe pump (Kent Scientific Co, Torrington, CT, USA). A 30 mL syringe was filled with saline and loaded into the pump. One liter of natural abundant ^129^Xe (~26%) was polarized up to 52% using the Xemed xenon polarizer and dispensed into a TedLar bag. A TedLar bag and a saline tube were connected to the membrane contactor (3M Liqui-Cel MM-0.5 × 1 Series) to mix HP Xe with saline. The outlet of the membrane contactor was connected to the 3.175 mm tube and the end of the tube was placed into the custom-built quadrature coil tuned to the Xe resonance frequency at 3T (35.33 MHz). A saline flow rate was set to 5 mL/min, 6 mL/min, 7 mL/min, and 10 mL/min. 

The ^129^Xe TOF GRE imaging was conducted using 20 TOF recovery times that varied from 200 ms to 2000 ms, with a step of 100 ms. The following parameters were used for imaging: TR/TE = 104.16 ms/1.22 ms, FA = 12.5°, FOV = 100 × 100 mm^2^.

### 2.3. In Vivo Cerebral Perfusion Measurements in Healthy Volunteers

This research study was approved (Jan. 28, 2019) by the research ethics boards (REB) at Lakehead University (LU) and the Thunder Bay Regional Health Sciences Centre (TBRHSC) (REB file number RP-307) and conducted in accordance with the Tri-Council Policy Statement-2 (TCPS-2). Nine healthy volunteers between the ages of 21 and 77 were recruited from the community for this study. All participants signed an informed consent form. All participants were cognitively normal and consented to the data obtained from them being disseminated in this report. 

#### 2.3.1. ^1^H Magnetic Resonance Imaging

Participants were placed into a dual tuned ^1^H/^129^Xe head coil in a Philips Achieva 3T clinical MRI scanner. T2-weighted ^1^H MRI were acquired using a turbo-spin echo (TSE) sequence with a Cartesian readout. The high-resolution anatomical proton images were acquired in axial and sagittal views. The sagittal images were acquired using a field-of-view equal to 250 × 250 × 46 mm^3^ and a voxel size of 0.98 × 1.04 × 6 mm^3^. The repetition time was set to 3 s and the echo time was equal to 80 s. The flip angle of excitation radiofrequency pulse was equal to 90°. Five slices were acquired, separated by 6 mm gaps. The axial scans were acquired using similar parameters. The only difference was in voxel size, which was equal to 0.98 × 0.99 × 6 mm^3^ in the axial scans.

#### 2.3.2. ^129^Xe Perfusion Mapping Using Time of Flight (TOF)

Enriched ^129^Xe (83%) was polarized to ~50% using a Xemed xenon polarizer and dispensed into 1 L Tedlar bags. The participant breathed in the gas and held their breath for 20 s. The dynamic ^129^Xe TOF imaging pulse sequence with a Cartesian gradient echo readout was initiated simultaneously with a breath-hold. Three dynamic projection brain images were acquired during the breath-hold. The TOF recovery delays were equal to 2.5 s, 6.7 s, and 7.1 s. The field of view of gradient echo pulse sequence parameters was equal to 250 × 250 mm^2^ and the acquisition matrix was set to 20 × 20. The excitation pulse flip angle was set to 20°. The repetition time was equal to 4 ms, the eco time was equal to 0.71 ms, and the bandwidth per pixel was 382 Hz.

To conduct hemodynamic response mapping, each participant was given two Xe MRI scans, one while staring at a gray screen (baseline) and another while exposed to an external stimulus, such as a flashing visual pattern (the visual stimulus used in this study can be downloaded from the link presented in the Supplementary Material S3) or s rapid left fist squeeze (motor stimulus). Exposure to the visual stimulus started approximately 7 s prior to image acquisition. Immediately prior to image acquisition, the participant inhaled 1 L of the HP ^129^Xe gas and held their breath for a period of 20 s.

A dynamic ^129^Xe TOF imaging pulse sequence with a Cartesian gradient echo readout was initiated simultaneously with a breath-hold using the following parameters: FOV = 250 × 250 mm^2^, TR/TE = 4 ms/0.71 ms, FA = 20°, BW = 382 Hz, acquisition matrix = 20 × 20. Three dynamic projection brain images were acquired during the breath-hold. The TOF recovery delays were equal to 2.5 s, 6.7 s, and 7.1 s for axial projections and 1 s, 6.5 s, and 7.1 s for the sagittal view. ^129^Xe TOF perfusion-weighted images were acquired in aial view with a slice thickness of 70 mm and in sagittal view with a slice thickness of 300 mm.

#### 2.3.3. ^129^Xe Perfusion Mapping Image Processing

Briefly, ^129^Xe images were zero-padded in k-space to a 32 × 32 matrix and a Fast Fourier transform (FFT) was applied to create MR images. SNR maps were created by dividing each pixel by the standard deviation of the noise region. Based on Killian’s model of dissolved Xe^18^, the mathematical theory was generalized and applied for HP ^129^Xe perfusion mapping (for a detailed explanation, see Supplementary Material S1). Three SNR maps created from TOF images and acquired with different recovery times were fitted pixel-by-pixel using the linear equation of the SNR delay time dependence (see Supplementary Material S2), and slope maps were created. The slope maps were further recalculated into perfusion maps using the developed model (see Supplementary Material S1). The SNR of the perfusion images was calculated as the ratio of the mean pixel intensity value from the brain region to the standard deviation of the selected square region from the background. The measured SNR values were compared to the average ASL SNR at 3T published in [39] using one-sample t-test. Xe hemodynamic response maps were created by subtracting the baseline slope map from the task slope map. An Xe functional brain map was overlaid on top of the high-resolution, T_2_-weighted proton image. Additional image processing information can be found in the Supplementary Material S4. 

#### 2.3.4. BOLD fMRI Image Acquisition

Participants were placed into an eight-channel Philips SENSE coil tuned to the ^1^H nucleus. 180 dynamic multi-slice echo-planar imaging (EPI) scans were acquired using the following parameters: FOV = 250 × 250 × 119 mm^3^, acquisition matrix = 64 × 64, voxel size = 3.91 × 3.91 × 4 mm^3^, TR/TE = 2 s/30 ms, FA = 90°, 24 slices with a 1 mm slice gap. Participants were subjected to the stimuli described above for the first 20 s and then a rest period for 20 s. Overall, 18 stimulus/rest repetitions were presented during the BOLD fMRI scans. 

For anatomical localization purposes, high-resolution, T_2_-weighted, axial images were acquired using a turbo-spin echo (TSE) pulse sequence using the following parameters: FOV = 250 × 250 × 119 mm^3^, voxel size = 0.98 × 0.99 × 4 mm^3^, TR/TE = 3 s/80 ms, FA = 90°, 24 slices of 4 mm thickness separated by 1 mm gaps.

#### 2.3.5. BOLD fMRI Image Processing

BOLD fMRI and T_2_-weighted anatomical images were converted from Philips PAR into Analyze format, and multivolume images were segmented into multiple 3D hdr files using MRIcro 1.40 (by Chris Rorden). The first 20 EPI scans (first complete cycle) were discarded to avoid T_1_ effects. fMRI data were processed using SPM12 [40] software using MATLAB R2018b (The Mathworks, Inc, Natick, MA, USA). Following manual alignment of the structural T_2_ image to the average canonical T_2_ image, the EPI image was manually aligned to the processed structural image. Using the SPM12 software, the obtained aligned EPI images were realigned and only the mean image was resliced. After slice timing and co-registration (an estimate), segmentation of the anatomical T_2_ image was performed and the final image was corrected to remove the spatially varying artifact (modulation of the image signal intensity). The functional and anatomical images were normalized using a saved deformation field with specification of the voxel size. Smoothing of the functional images was done with Full Width at Half Maximum (FWHM) set to 6 mm in all directions. The positive t-contrast (stimulus > rest) was calculated for the final functional image. The *p*-value was adjusted to 0.05 with a Family-Wise Error (FWE) correction. No masking or thresholding were applied during the image processing. Statistical maps were overlaid on volume-rendered brain images provided by SPM12.

Signal enhancement was estimated using the MarsBar extension for SPM12 (by Matthew Brett, available at http://marsbar.sourceforge.net/). The region of interest (ROI) was specified for the cluster with the highest intensity from SPM results file. This was viewed and exported as an image with the base space. 

## 3. Results

### 3.1. In Vitro Evaluation of HP ^129^Xe TOF Pulse Sequence

The ^129^Xe TOF pulse sequence (Figure 1) was programmed on a Philips 3.0T Achieva MRI scanner and tested using a flow phantom (Figure 2a). A previously developed model of Xe uptake in the brain [41] was modified to facilitate quantitative perfusion measurements using ^129^Xe TOF imaging. The complete analytical function, which describes the signal evolution in a brain voxel (see Supplementary Material S1), is difficult to implement practically and, therefore, a simplified model for the ^129^Xe wash-in phase was developed by employing several assumptions. First, ^129^Xe relaxation in blood [32,41,42,43,44,45] is the predominant factor of polarization decay during the wash-in phase. Second, the value of the sum of the tissue perfusion-to-partition coefficient ratio plus the relaxivity of HP ^129^Xe in the tissue is small. With these two assumptions, the SNR evolution during the TOF imaging sequence can be expressed as a linear function (for the detailed mathematical derivation please see Supplementary Information), where the slope of the line is directly proportional to the sum of perfusion of all tissues in the voxel. This approach is valid only for short recovery times and can be easily used in practice, albeit yielding an underestimation of the slope of the TOF recovery curves for longer TOF delay times.

The flow rates were set to 5 mL/min, 6 mL/min, 7 mL/min, and 10 mL/min. The signal-to-noise ratio (SNR) increased linearly at small recovery times, followed by a nonlinear transition (intermediate recovery times) up until saturation (long recovery times) (Figure 2b). The nonlinear dynamic at intermediate recovery times can be explained by a nonlinear ^129^Xe velocity distribution in saline flow cross-sections. The signal became saturated earlier for the fastest flow rate, whereas a slight signal saturation was observed for the slowest flow rate. The slopes of the TOF recovery curves were calculated, and a strong Pearson’s correlation (r = 0.988, *p* < 0.05) between the flow rate and the signal recovery rate was observed (Figure 2c). A difference of 1 mL/min in flow caused the observed change in the recovery rate. 

### 3.2. HP ^129^Xe TOF Cerebral Perfusion Imaging

Human imaging experiments were approved by the research ethics boards at Lakehead University and the Thunder Bay Regional Health Science Centre and conducted in accordance with the Tri-Council Policy Statement 2. All participants signed an informed consent form. During human imaging, the changes in the volunteers’ blood oxygen saturation levels were within the normal range. All images were acquired during a single breath-hold after inhalation of 1 L of HP ^129^Xe. The obtained images were converted from radiological to anatomical views.

**Figure 3:**
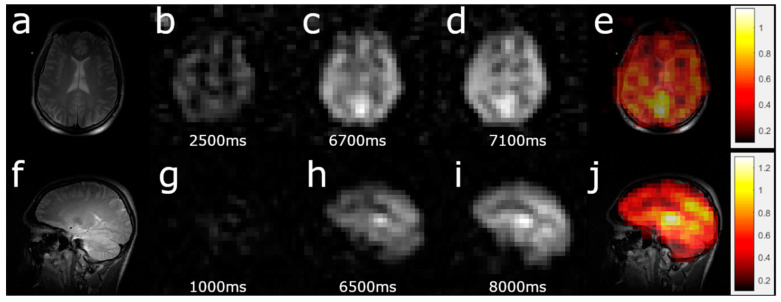
Example of perfusion map acquisition. (**a**,**f**) High-resolution, T_2_-weighted ^1^H scans for brain localization. (**b**–**d**) Three dynamic HP ^129^Xe TOF images acquired 2.5 s, 6.8 s, and 7.1 s after the application of a depolarization radiofrequency pulse in the axial projection. The image artifact in the top left corner in **b** is from excess gaseous ^129^Xe at the end of the inhalation tube connected to the TedLar bag. The gradual signal-to-noise ratio increase can be observed with increasing wash-in time. The slope map was created by a pixel-by-pixel linear fit of the ^129^Xe brain images. (**e**) The perfusion map (measured in mL of blood per mL of tissue per min) created by the pixel-by-pixel recalculation of the TOF slope was used to calculate the sum of the perfusion rates of gray and white matter superimposed on top of a high-resolution proton brain image. The calculated values of perfusion agreed with previously observed values [41,46]. (**g**–**i**) Three dynamic TOF images acquired after 1s, 6.5 s, and 8 s TOF in the sagittal view. (**j**) Perfusion map in the sagittal view. Similar to **e**, the intensity values were the net sum of the white and gray matter perfusion rates.

Figure 3a,f demonstrate the high-resolution, T_2_-weighted proton images which were used for brain localization. Figure 3b–d show ^129^Xe TOF images acquired in the axial view after TOF of 2.5, 6.5, and 7.1 s. HP ^129^Xe TOF sagittal images (Figure 3g–i) were acquired following 1 s, 6.5 s, and 8 s TOF delays. Following pixel-by-pixel calculations of the TOF slope, the corresponding ^129^Xe TOF perfusion-weighted images were reconstructed in both the axial and sagittal projections. The average SNR of the reconstructed, perfusion-weighted images was equal to 11.2 ± 2.9 (in the sagittal projection) and 9.5 ± 2.9 (in the axial projection). Using the theoretical calculations shown in the Supplementary Information, the slope maps were transformed into net perfusion maps (Figure 3e,j). Although the ^129^Xe TOF pulse sequence is similar to the ASL pulse sequence, the manipulation of the magnetization is fundamentally different. The contrast of perfusion-weighted ASL images comes from the difference between control and spin-tagged images, whereas the contrast of TOF perfusion maps comes directly from the velocity of incoming blood flow.

### 3.3. HP ^129^Xe Hemodynamic Response Detection

**Figure 4:**
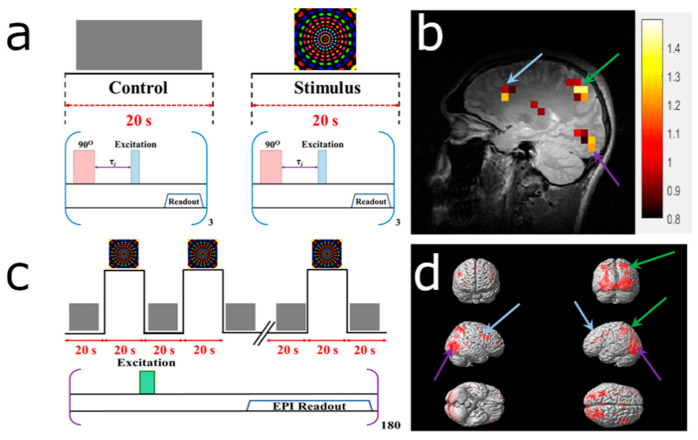
Detection of a hemodynamic response from a colorful visual stimulus using HP ^129^Xe perfusion mapping validated by blood oxygenation level-dependent (BOLD) functional brain MRI (fMRI). (**a**) Experimental design used for hemodynamic response detection. Two separate perfusion maps were acquired during the control (gray screen) and visual stimulation. (**b**) Hemodynamic response map created by subtracting the control perfusion map from the stimulated perfusion map and overlaid on top of a high-resolution proton scan. Activation of the occipital lobe, superior parietal lobe, and frontal gyrus was observed. (**c**) BOLD fMRI experimental design for validation of the HP ^129^Xe technique. (**d**) BOLD fMRI 3D activation maps demonstrate a correlation with a ^129^Xe hemodynamic response map. The activated areas are indicated by colored arrows. The matched activated areas on the Xe image are indicated with arrows of the corresponding color.

**Figure 5:**
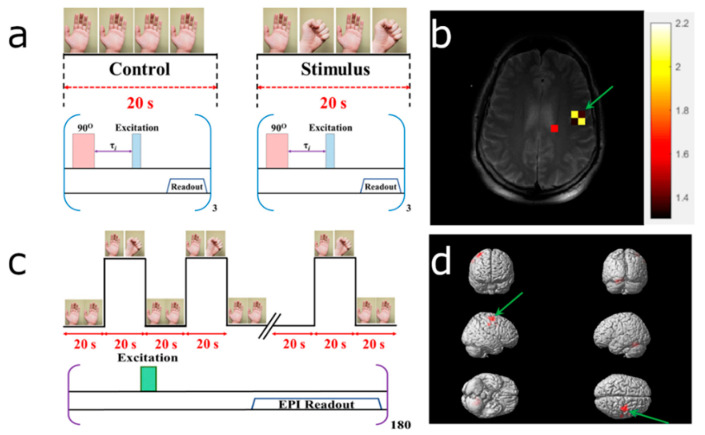
Detection of the hemodynamic response to a motor stimulus using HP ^129^Xe perfusion mapping corroborated by blood oxygenation level-dependent (BOLD) functional brain MRI (fMRI). (**a**) Experimental design used for hemodynamic response detection. Two separate perfusion maps were acquired during the control (calm rest) and motor stimulation (left fist clenching) stages. (**b**) Hemodynamic response map created by subtracting the control perfusion map from the stimulated perfusion map and overlaid on top of the high-resolution proton scan. Activation of the right posterior precentral gyrus (i.e., the motor cortex) was observed. (**c**) BOLD fMRI experimental design for validation of the HP ^129^Xe technique. (**d**) BOLD fMRI 3D activation maps. Activation was observed from the motor cortex (green arrow); this result correlated with the HP ^129^Xe hemodynamic response map.

The blood-flow changes due to the hemodynamic response (HDR) to visual and motor stimuli (see Supplementary Material S3) ertr detected using ^129^Xe TOF perfusion imaging. The experimental designs for HP ^129^Xe TOF and ^1^H BOLD fMRI are shown in Figure 4a,c and Figure 5a,c. The reconstructed HDR maps nicely correlated with ^1^H BOLD fMRI and demonstrated the activation of the same brain regions. Activation areas demonstrated on ^129^Xe HDR maps agreed with previously published results [47,48,49,50]. 

Figure 4 illustrates the results obtained after subjecting the volunteer to the colorful rotating dotted visual stimulation. HP ^129^Xe TOF detected activation of the visual cortex (occipital lobe), superior parietal lobe, and frontal gyrus (Figure 4b). These results completely agreed with ^1^H BOLD fMRI images acquired by ^129^Xe TOF image (Figure 4d).

Figure 5 illustrated the experimental design (a,c) and obtained ^129^Xe TOF HDR maps (b) and ^1^H BOLD fMRI images (d) of the representative volunteers subjected to the motor stimulus. Both approaches detected activation of the motor cortex contralaterally to the clenched fist.

## 4. Discussion

There is no HP MRI approach currently available which is suitable for quantitative perfusion imaging. Recently, HP MRI was used to detect cerebral perfusion changes caused by stroke [35] and Alzheimer’s disease [34]. Since HP ^129^Xe freely dissolves in the blood and travels to highly perfused organs, it can be used as a contrast agent for quantitative imaging of perfusion and blood flow. Therefore, HP ^129^Xe TOF blood flow mapping is a possible next step in blood flow and perfusion measurement.

Previous works focused on the dynamic acquisition of multiple brain projection images during a breath-hold and the following wash-out time of ^129^Xe from the brain [6,33,34]. Acquired images were subsequently averaged [6,33] or postprocessed to characterize the wash-out process of ^129^Xe from the brain [34]. These approaches were strongly dependent on variations in the breath-hold period and the individual lung capacities of the subjects. There is typically some signal variation in the ^129^Xe images acquired from the same subject from different breath-holds due to individual variation in the amount of inhaled gas, the amount of gas actually in the alveolar lung space, and the amount of gas remaining in the trachea and larynx. Therefore, a proper comparison of images acquired from different breath-holds is challenging and requires proper signal normalization.

The HP ^129^Xe TOF technique is the first differential HP MRI technique, to our knowledge, that relies on ^129^Xe signal recovery rate calculation. This imaging approach is fundamentally different from all other MRI methods for blood flow mapping and relies on the special properties of HP MRI. An initial depolarization radiofrequency pulse applied to the imaging region provides the necessary reference for accurate imaging and makes the obtained perfusion-weighted images independent of individual lung capacity and breath-hold variations. In addition, the HP ^129^Xe TOF approach allows quantitative perfusion mapping using the derived theory (see Supplementary Material S1).

Although HP ^129^Xe blood flow mapping is similar to ASL in terms of pulse sequences, this technique overcomes the issue of low signal-to-noise ratio, which is typical for ALS perfusion images (the mean SNR of the Xe perfusion maps was approximately double compared to values acquired for ASL [39] (*p* < 0.05)) [1,51]. The obtained ^129^Xe perfusion maps (Figure 3e,j) looked similar to results previously obtained from healthy volunteers acquired using ASL MRI [52]. With the additional separation of the HP ^129^Xe signals originating from various brain tissues from the signal of ^129^Xe dissolved in blood (either via different phase separation methods or using chemical shift selective imaging), it should be possible to quantitively map the perfusion of each tissue in a region of interest. Signal separation of the dissolved ^129^Xe phases may allow the use of an exact mathematical solution for perfusion map calculations (see Supplementary Material S1), which would eliminate any potential underestimation of perfusion caused by using an approximate linear model. 

Recently, multiple advances in ASL perfusion imaging were achieved by developing a pseudo-continuous ASL (pCASL), resulting in an SNR increase compared to conventional ASL [16,17,18,53]. pCASL perfusion images have higher spatial resolution compared to the present HP ^129^Xe TOF images, mainly due to their significantly better developed hardware (multichannel coils used for parallel imaging during perfusion imaging) and software available for conducting proton ASL MRI. By using a parallel imaging approach, compressed sensing, nonCartesian k-space trajectories, and by developing multichannel ^129^Xe coils, it should be possible to acquire HP ^129^Xe perfusion maps with a spatial resolution comparable to pCASL resolution and with a significantly higher SNR. 

Although the scan time for acquiring a single brain perfusion map was ~20 s, it could be shortened to ~10 s by reducing TOF recovery time delays. The scan time could be shortened further for kidney perfusion measurements, since the reasonably high SNR of HP ^129^Xe dissolved in the kidneys can be acquired in less than 4 s [35]. 

As one practical application, the HP ^129^Xe perfusion mapping technique could be used for functional imaging of the human brain, which would allow direct hemodynamic response mapping. HP ^129^Xe functional brain maps can be acquired significantly faster compared to traditional BOLD functional images. In addition, this approach requires less postprocessing than the BOLD technique, which makes it less ambiguous in terms of data processing. Although BOLD fMRI and HP ^129^Xe perfusion mapping are two fundamentally different techniques and quantitative comparisons between them are challenging, the estimated percent signal enhancement of the functional maps obtained by HP ^129^Xe perfusion mapping was up to two orders of magnitude larger than the BOLD fMRI images. Xenon gas is used in clinics as a general inhalation anesthetic with continuous breathing over several minutes at a total lung concentration greater than 50% [54]. Previous studies demonstrated an acceptably low incidence of adverse effects caused by inhalation of xenon with an overall alveolar concentration below 32% [55]. The concentration of inhaled HP ^129^Xe in our study was 20–25% (considering a lung volume of 4–5 L), and there were no side effects observed after multiple inhalations and breath-holds.

A major limitation of the approach demonstrated for ^129^Xe TOF functional imaging is the requirement of acquiring projection images instead of multislice images due to the limited quantity of HP ^129^Xe that is transported to the brain. This issue can be overcome by using parallel imaging and compressed sensing techniques, which allow for faster acquisition with higher SNR per TOF image. Therefore, a larger number of slices can be scanned during a single breath-hold. Another limitation of HP ^129^Xe perfusion mapping, in terms of its clinical relevance, is the ability of a participant to hold their breath. Although the short scan time required allows perfusion mapping in patients with lung diseases who can only tolerate short breath-hold periods, this technique is not possible for participants who cannot hold their breath for at least eight seconds. As an alternative, continuous breathing of an HP ^129^Xe mixture with oxygen could be considered. A continuous breathing protocol has so far only been utilized for animal lung studies [56,57]. Translation of this breathing protocol to HP perfusion imaging might be challenging due to the oxygen-induced T_1_ shortening of HP ^129^Xe and subsequent decrease in the ^129^Xe MRI signal. On the other hand, this approach could allow for multiple signal averaging and expand the application of HP ^129^Xe TOF perfusion imaging. As a particular example, patients who are not able to hold their breath for eight seconds could be scanned using continuous breathing. Moreover, this might allow for HP ^129^Xe perfusion imaging during clinical procedures when patients are under HP ^129^Xe-induced anesthesia. Since the ^129^Xe TOF technique is based on selective depolarization of ^129^Xe nuclei, the MRI scanner needs to be properly calibrated, otherwise there will be an additional contribution to the signal from partially depolarized nuclei. Another potential limitation for some HP perfusion imaging applications is the time needed for ^129^Xe polarization. The polarizer used in this study requires about 15 min to polarize 1 L of ^129^Xe. Therefore, as a diagnostic tool, HP ^129^Xe TOF might not be suitable for urgent imaging purposes. On the other hand, Xe was shown to demonstrate neuroprotective properties [58], indicating a potential use of this modality for cerebral perfusion imaging in stroke patients, for example. Finally, the other limitations of this technique are the high cost of enriched ^129^Xe gas and the polarizer instrument. It should be noted that the use of enriched ^129^Xe is required by an overall low HP ^129^Xe signal in the brain. With further hardware development, the use of naturally abundant ^129^Xe, which is substantially less expensive, is expected to become more feasible. 

Overall, HP ^129^Xe perfusion mapping is a novel HP MRI method which, we believe, could expand future knowledge in the field of perfusion imaging and functional brain mapping. It may also be a useful tool for the detection of perfusion changes in different organs and for further diagnostics regarding perfusion-related diseases. 

## 5. Patents

Based on the results of this work, the provisional patent entitled “Method to Detect Brain Functional Activities Using Hyperpolarized ^129^Xe MR” was filed.

## Supplementary Materials

The following are available online at https://www.mdpi.com/2075-4418/10/9/630/s1.
