# Supplementary material for: Hyperpolarized 129Xe Time-of-Flight MR Imaging of Perfusion and Brain Function
Source: Diagnostics (Basel). 2020 Aug 25;10(9):630. doi: 10.3390/diagnostics10090630 (PMC7554935; doi:10.3390/diagnostics10090630)
Supplement: Supplementary file 1 [file diagnostics-10-00630-s001.zip › Supplementary information Hyperpolarized Xe imaging of perfusion and brain function.docx]

Hyperpolarized ^129^Xe Time-of-Flight MR imaging of perfusion and brain function

Yurii Shepelytskyi ^1, 2^, Francis T. Hane ^2, 3^, Vira Grynko ^1, 2^, Tao Li ^3^, Ayman Hassan ^4,5^, Mitchell S. Albert ^2, 3, 5, *^

**S1. Theoretical aspect of HP ^129^Xe perfusion imaging**

Due to the solubility of ^129^Xe in blood^1^, the ^129^Xe travels with the blood stream to the organs. Following Kilian’s^2^ model, the time evolution of the ^129^Xe concentration within tissue obeys the differential equation:

$$\frac{dC_{i}\left(t\right)}{dt}=F_{i}\left(C_{B}\left(t\right)-\frac{1}{p_{iB}}C_{i}\left(t\right)\right)-\frac{C_{i}\left(t\right)}{T_{1i}}$$

where C_i_ is a HP ^129^Xe concentration within the tissue i, F_i_ is a perfusion rate, p_iB_, the partial coefficient between the tissue i and blood, T_1i_ spin-lattice relaxation time of HP ^129^Xe within the tissue i. Kilian’s model can be expanded to describe the concentration of HP ^129^Xe in a one image voxel. We consider a case of the brain imaging. We assume that the MRI signal caused by HP ^129^Xe dissolved in white matter (WM), gray matter (GM) and blood. The concentration change in the one voxel can be expressed using the following system of equations:

$$\left\{\begin{matrix} \frac{dC_{W}\left(t\right)}{dt}=F_{W}\left(C_{B}\left(t\right)-\frac{1}{p_{WB}}C_{W}\left(t\right)\right)-\frac{C_{W}\left(t\right)}{T_{1W}} \\ \frac{dC_{G}\left(t\right)}{dt}=F_{G}\left(C_{B}\left(t\right)-\frac{1}{p_{GB}}C_{G}\left(t\right)\right)-\frac{C_{G}\left(t\right)}{T_{1G}} \\ C_{B}\left(t\right)=\lambda_{B}C_{A,0}e^{-t\left(\frac{1}{T_{1A}}+\lambda_{B}\frac{Q}{V_{A}}\right)}e^{-\frac{t_{B}}{T_{1B}}} \end{matrix}\right.$$

where T_1A_ is a spin-lattice relaxation time in the lungs; C_B,_ the ^129^Xe concentration reaching the image voxel at time t; t_B_, the time required for the blood to reach brain; λ_B_, the Oswald solubility of ^129^Xe in blood; T_1B_, ^129^Xe relaxation in blood; Q, pulmonary blood flow; V_A_, alveolar volume. The first two equations are inhomogeneous differential equations of the first order. These equations can be solved using the Lagrange’s variation of parameters method. The solution of homogeneous differential equation for the ^129^Xe concentration in white matter in which the integration constant was substituted by the time function is:

$$C_{W}=C_{0}(t)e^{-\beta_{W}t}$$

The C_0_(t) can be determined after substitution of (S.3) into correspondent equation of (S.2):

$$C_{0}(t)=\frac{\xi F_{W}}{\beta_{W}-\gamma}e^{t\left(\beta_{W}-\gamma\right)}+C^{*}$$

where

$$\beta_{W}=\frac{F_{W}}{p_{WB}}+\frac{1}{T_{1W}};\xi=\lambda_{B}C_{A0}e^{t_{B}\left(\frac{1}{T_{1A}}-\frac{1}{T_{1B}}+\lambda_{B}\frac{Q}{V_{A}}\right)};\gamma=\frac{1}{T_{1A}}+\lambda_{B}\frac{Q}{V_{A}}$$

The integration constant C* can be calculated since we know that there is a time (t_B_) that ^129^Xe needs to reach the brain. This gives the following condition:

$$C_{W}(t_{B})=0\Rightarrow C_{0}(t)=0$$

Substituting (S.4) into (S.6), the integration constant C* can be obtained. The Xe concentration time dependence in WM can be written as follows:

$$C_{W}\left(t\right)=\frac{F_{W}\xi}{\beta_{W}-\gamma}e^{-\gamma t}\left(1-e^{-\left(t-t_{B}\right)\left(\beta_{W}-\gamma\right)}\right)$$

Using the same approach, the concentration of Xe in GM can be expressed as follows:

$$C_{G}\left(t\right)=\frac{F_{G}\xi}{\beta_{G}-\gamma}\left(1-e^{-\left(t-t_{B}\right)\left(\beta_{G}-\gamma\right)}\right)$$

Based on the definition of the partial coefficient, the Xe concentration in blood can be written as

$$C_{B}=\frac{C_{W}+C_{G}}{p_{GB}+p_{WB}}$$

The net concentration of Xe in the image voxel can be described by the following equation:

$$\begin{matrix} C_{Voxel}=C_{B}+C_{G}+C_{W} \\ C_{Voxel}=\left(1+\frac{1}{p_{WB}+p_{GB}}\right)\xi e^{-\gamma t}\left(\frac{F_{G}}{\beta_{G}-\gamma}\left(1-e^{-\left(t-t_{B}\right)\left(\beta_{G}-\gamma\right)}\right)+\frac{F_{W}}{\beta_{W}-\gamma}\left(1-e^{-\left(t-t_{B}\right)\left(\beta_{W}-\gamma\right)}\right)\right) \end{matrix}$$

Equation S.10 describes both wash-in and wash-out of HP ^129^Xe into and out of the voxel. To obtain a more accurate blood flow map, equation S.10 should be corrected with respect to receiving bandwidth and used for pixel-by-pixel fit of ^129^Xe TOF images. However, the fitting process becomes ambiguous due to symmetry of equation S.10. Indeed, converting F_G_ into F_W_ and β_G_ into β_W_ yields to the same function. Therefore, model simplifications are needed for data analysis. Considering wash-in phase and assuming that relaxation in blood is predominant factor of signal decay, the following simplification can be used:

$$\gamma\to0;e^{-\gamma t}\to1$$

Second, we consider a small sum of tissue perfusion and relaxivity of ^129^Xe in tissue:

$$\frac{F_{i}}{p_{iB}}+\frac{1}{T_{1i}}<<1; 0<\beta<<1\Rightarrow1-e^{-\beta_{i}\left(t-t_{B}\right)}\approx\beta\left(t-t_{B}\right)$$

Using assumptions (S.11) and (S.12), the equation S.10 can be rewritten as:

$$C_{Voxel}=\left(1+\frac{1}{p_{WB}+p_{GB}}\right)\xi\left(F_{G}+F_{W}\right)\left(t-t_{B}\right)$$

Equation S.13 is a linear function of time. The signal to noise ratio is proportional to the concentration of the nuclei (SNR=const∙C_Voxel_). Therefore, the signal to noise ratio evolution during the ^129^Xe TOF scans can be written as:

$$SNR=SNR_{0}\left(F_{G}+F_{W}\right)\left(\tau-t_{B}\right)$$

The slope of equation S.14 is a scaled sum of perfusion rates of WM and GM. This equation can be used for creation of perfusion-weighted images of the human brain. By using similar approach, it is possible to analyse perfusion of any other organ.

The solution of the Eqn. S.1 can be written as:

$$\begin{matrix} C_{i}\left(t\right)=\frac{F_{i}\xi}{\beta_{i}-\gamma}e^{-\gamma t}\left(1-e^{-\left(t-t_{B}\right)\left(\beta_{i}-\gamma\right)}\right); \\ \beta_{i}=\frac{F_{i}}{p_{iB}}-\frac{1}{T_{1i}};\xi=\lambda_{B}C_{A0}e^{t_{B}\left(\frac{1}{T_{1A}}-\frac{1}{T_{1B}}+\lambda_{B}\frac{Q}{V_{A}}\right)};\gamma=\frac{1}{T_{1A}}+\lambda_{B}\frac{Q}{V_{A}} \end{matrix}$$

Considering N tissues in voxel, the net ^129^Xe concentration can be expressed as follows:

$$C_{Tot}(t)=\sum_{i=1}^{N} C_{i}\left(t\right)+C_{B}\left(t\right)$$

And the SNR evolution with TOF recovery obeys the following equation:

$$SNR\left(\tau\right)=SNR^{'}\left(1+\frac{1}{\sum_{i=1}^{N} p_{iB}}\right)\xi e^{-\gamma\tau}\sum_{i=1}^{N} \frac{F_{i}}{\beta_{i}-\gamma}\left(1-e^{-\left(\tau-t_{B}\right)\left(\beta_{i}-\gamma\right)}\right)$$

Eqn. S.17 can be further simplify using similar to S.12 approach.

S2. **Hemodynamic response detection using HP ^129^Xe perfusion imaging**

During the brain stimulation, there is an increased blood flow to the activated brain region^3^. This phenomenon is known as hemodynamic response. It yields an increase in perfusion in the activated brain area. Therefore, in the stimulated brain areas, the slope of equation S.14 is expected to be higher compared to the slope measured from the same brain region without stimulation. The subtraction of the resting slope map from the TOF slope map acquired during the brain stimulation yields the ^129^Xe hemodynamic response map. This map can be interpreted as a functional image of the brain due to correlation between hemodynamic response and the brain activity.

**S3. Visual Stimulus:**


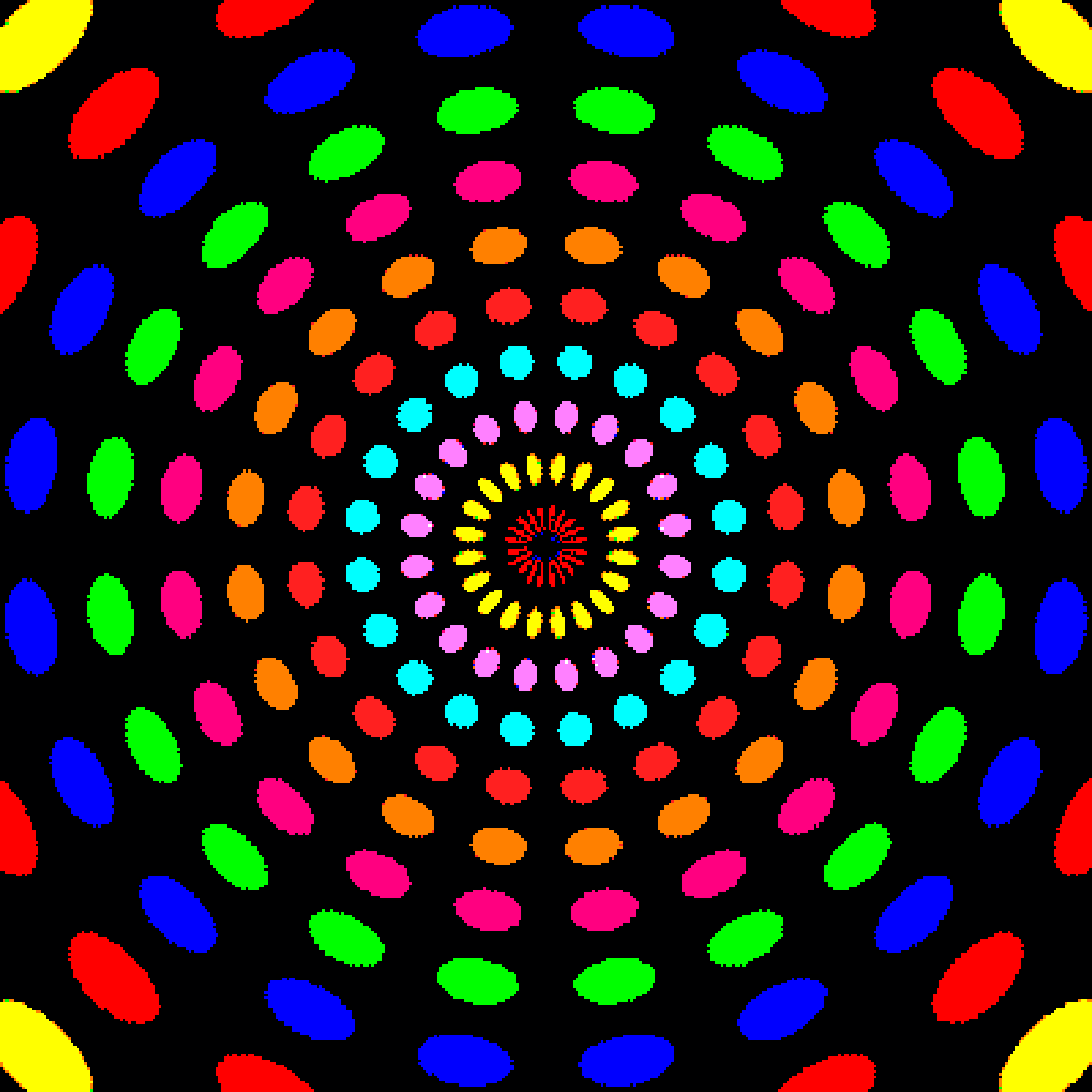


The gif file can be downloaded from the following link:

<http://samlib.ru/img/m/marina_maleshewich/coloured_minds-2/dvizhenie1.gif>

**S4. Image Postprocessing Procedure:**

| **TOF control scan** | **TOF stimulated scan** |
| --- | --- |
| 1. Acquire control (gray screen) and stimulated (flashing dots) TOF scans using the following recovery times: 1 s, 6.5 s and 8 s. | |
| 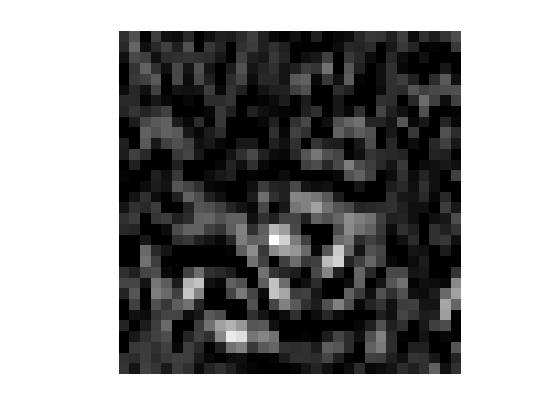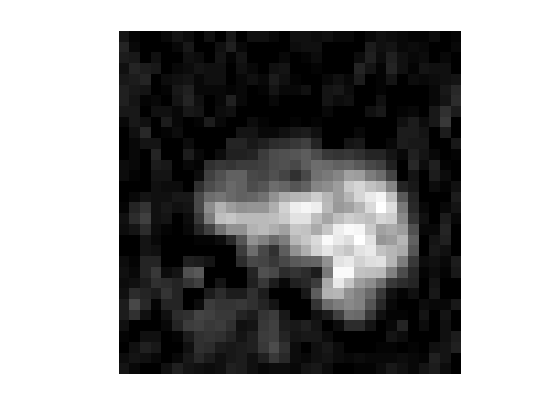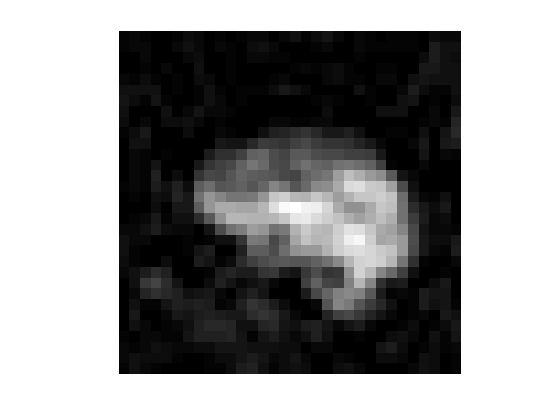 | 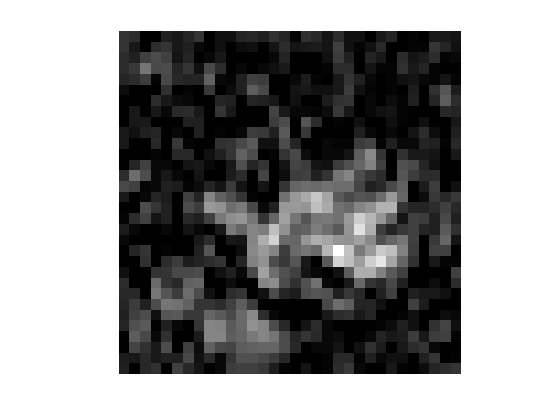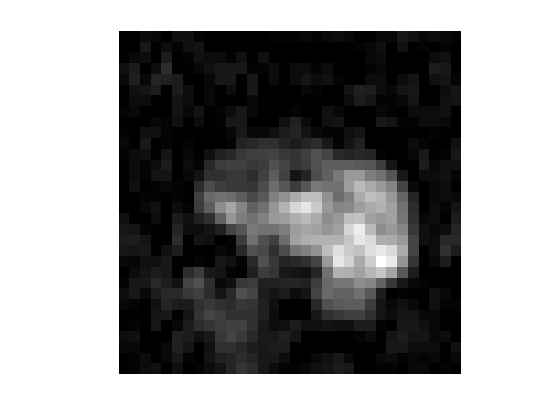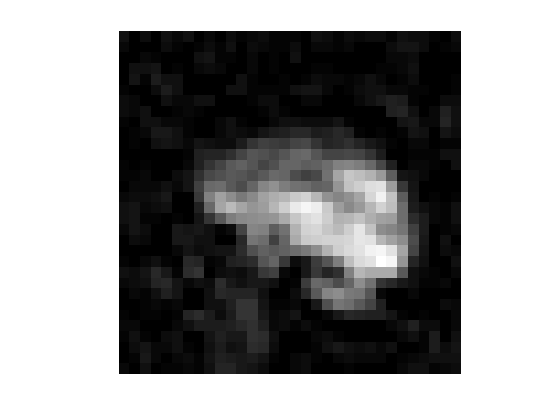 |
| 2. Analyzing the images using the Matlab Script attached below. Select the ROI on a control scan first (region which contains the brain). | |
| 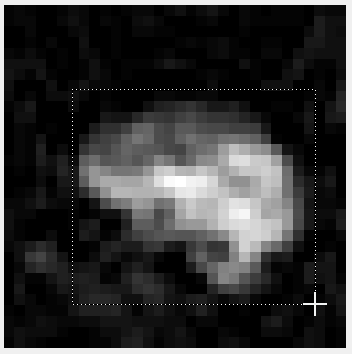 |  |
| 3. Select the noise regions on the control and stimulated images | |
| 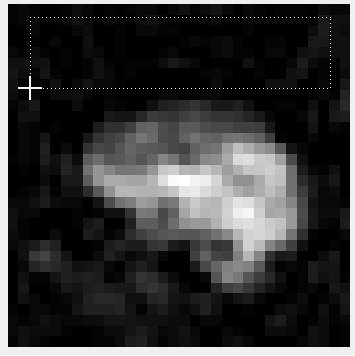 | 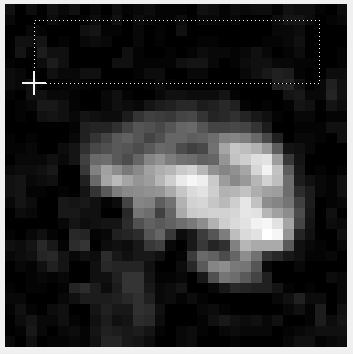 |
| 4. The TOF perfusion-weighted images for rest state and stimulus will be calculated. Each pixel of each image is divided by standard deviation of the noise to obtain the SNR images. | |
| 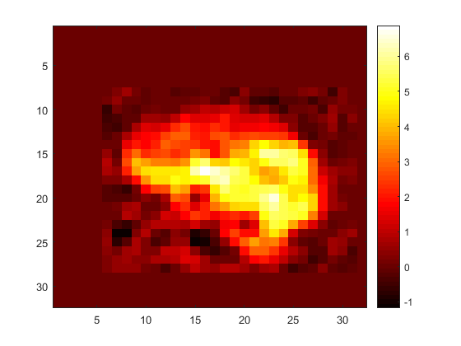 | 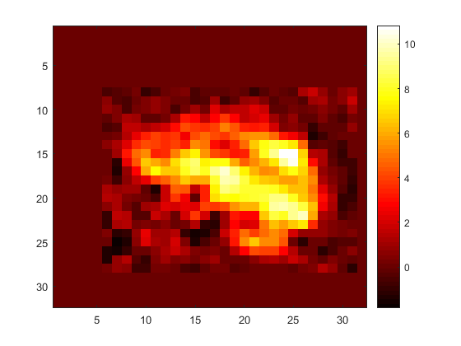 |
| 5. Subtraction yields hemodynamic response map | |
| 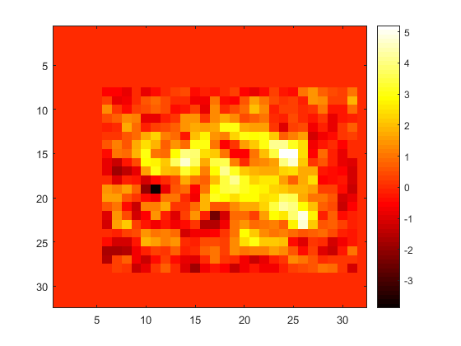 | |
| 6. After thresholding for statistical significance, the clear functional brain map is created | |
| 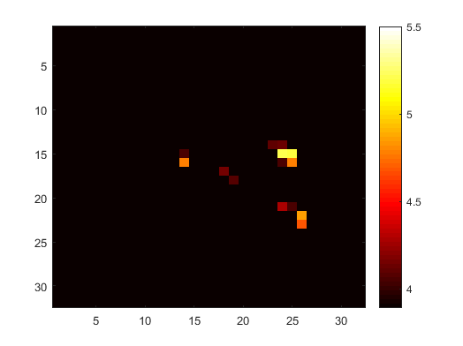 | |
| 7. The functional brain map can be superimposed on top of a high resolution anatomical MRI to localize the activated brain regions | |
| 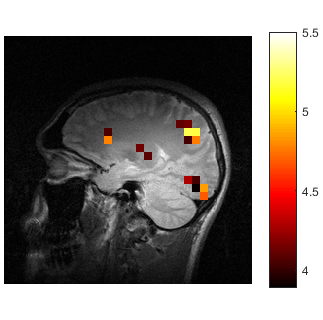 | |

**References**

1. Zhou X, Mazzanti ML, Chen JJ, et al. Reinvestigating hyperpolarized 129Xe longitudinal relaxation time in the rat brain with noise considerations. *NMR Biomed*. 2008;21(3):217-225.

2. Kilian W, Seifert F, Rinneberg H. Dynamic NMR Spectroscopy of Hyperpolarized 129Xe in Human Brain Analyzed by an Uptake Model. *Magn Reson Med*. 2004;51(4):843-847. doi:10.1002/mrm.10726

3. Ogawa S, Lee TM, Nayak AS, Glynn P. Oxygenation-sensitive contrast in magnetic resonance image of rodent brain at high magnetic fields. *Magn Reson Med*. 1990;14(1):68-78. doi:DOI 10.1002/mrm.1910140108
